# Resistance gained, resistance lost: An explanation for host–parasite coexistence

**DOI:** 10.1371/journal.pbio.3000013

**Published:** 2018-09-24

**Authors:** Britt Koskella

**Affiliations:** Department of Integrative Biology, University of California, Berkeley, Berkeley, California, United States of America

## Abstract

Host populations are under continual selection by parasites due to reduced fitness of infected individuals relative to uninfected individuals. This should select for host resistance against parasites, and ample evidence from the laboratory and natural populations demonstrates that hosts can respond rapidly to parasitism by evolving resistance. Why then do parasites still exist? In part, this is due to ongoing arms races as parasites evolve counteradaptations to overcome resistance and to the presence of spatial structure and refuges. However, host–parasite coexistence can also be explained through loss of resistance over time due either to selection against costly resistance mechanisms or constant loss of resistance via reversion mutations.

## Abstract 2

This Primer explores the ways in which host resistance to parasites can be lost, and describes a new study suggesting that the continual loss of resistance might be an important mechanism for the maintenance of bacteriophages in nature.

The evolution of host resistance against parasites (used broadly here to include any organism that lives in or on another organism at the expense of the latter) has been a long-standing focus of research in biology. This is in part because understanding host resistance allows us to better predict the spread of disease in human, agricultural, and natural populations but also because parasite-mediated selection has been proposed and supported as a general mechanism to explain diversity [1–3]. Parasites act as an important and ubiquitous evolutionary force, constantly shaping the evolution of their host populations. Because parasites inherently cause harm to their hosts, intuition would suggest that any mechanism by which a host can avoid or resist infection should be selected for over time. Indeed, the evolution of resistance to parasites has been well documented both in controlled laboratory studies [e.g., 4, 5] and in studies of natural populations [e.g., 6, 7].

Two of the most well-understood examples of host resistance are selection for R-genes in plant populations [8], in which the presence of pathogens with matching avirulence genes leads to a defense response such as localized host cell death, and the rapid evolution of bacterial resistance against phages. The latter typically occurs through either constitutive immunity such as receptor modification, in which the binding site of phages is lost or altered [9], or via innate/adaptive immunity such as restriction modification (RM) and clustered regularly interspaced short palindromic repeats (CRISPR) systems [10–12] (although note that a plethora of other bacterial resistance mechanisms have been described [13]). In both cases, there exists good evidence that resistance is specific, such that it protects hosts against particular parasite species or even strains and that polymorphisms at these resistance loci are common [8,14], suggesting ongoing and often temporally or spatially variable selection pressure.

Less well studied is the loss of host resistance from populations over time. Studies of natural populations rarely find resistance to be a fixed trait either within or across populations [8,15], but why this would be the case despite the advantage of resistance is not always clear. Loss of resistance can result from at least three mechanisms (Fig 1). In the first case, host resistance can be lost over time not due to host evolution but rather due to parasite counteradaptation. For example, the Recognition of *Peronospora parasitica* 13 (*RPP13*) resistance gene of *Arabidopsis thaliana* that confers protection against the causal agent of Powdery Mildew, *Hyaloperonospora parasitica*, is known to be highly polymorphic. As would be predicted if this diversity was the result of ongoing host–pathogen coevolution due to continual selection pressure for pathogens to overcome new host resistance alleles and vice versa; a similarly high level of amino acid polymorphism has been observed for the pathogen’s matching avirulence gene, *Arabidopsis thaliana* Recognized 13 (*ATR13*) [8]. Similarly, the activity of CRISPR-Cas bacterial immunity against phages has been shown to be overcome by some phages as a result of an acquired phage “anti-CRISPR” system [16]. In the ongoing arms race between a host and its parasite, resistance is often only a temporary phenotype.

**Fig 1:**
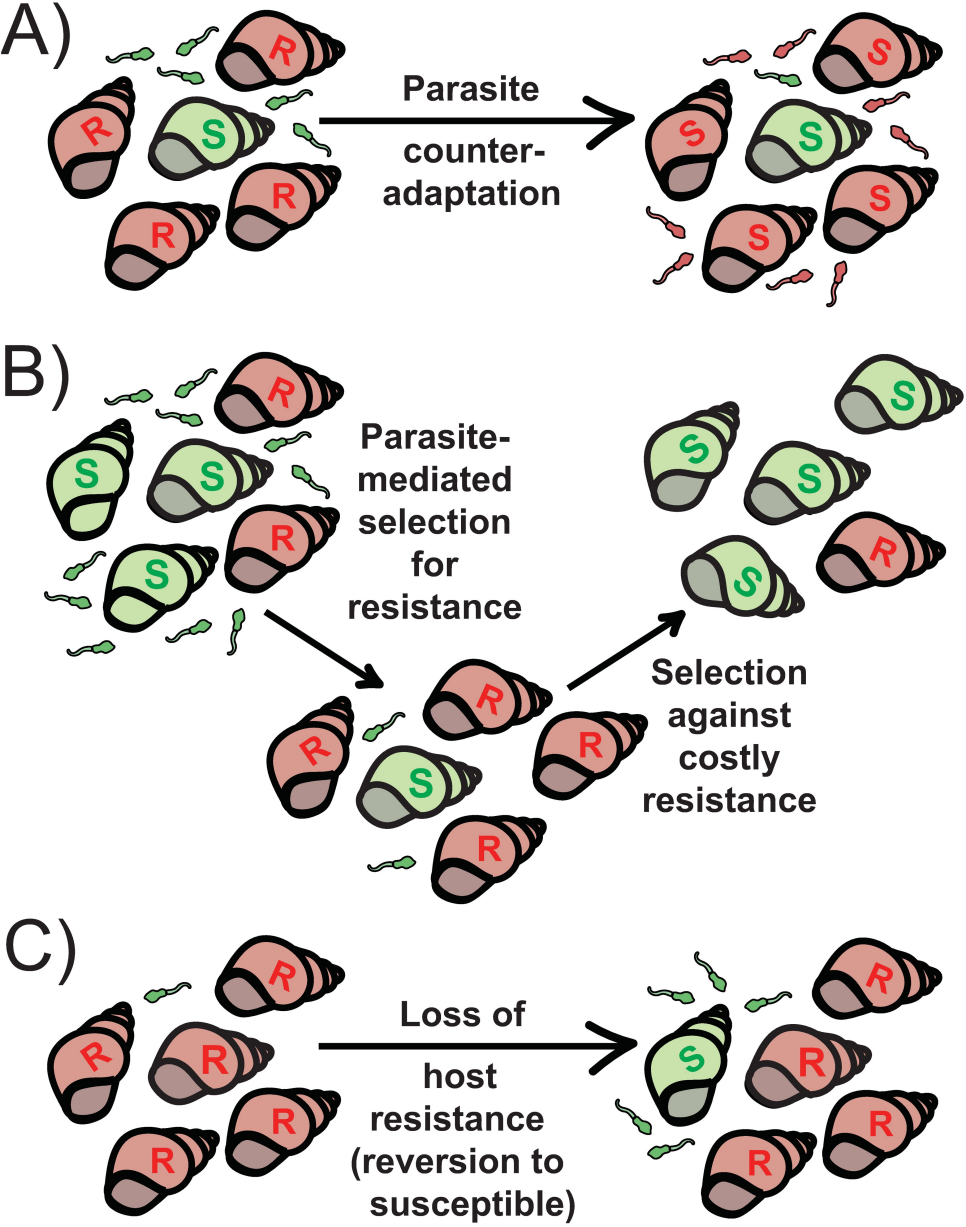
Illustration of the proposed mechanisms by which resistance can be lost from host populations over time, using snails and their trematode parasites. A) Resistance can be lost if parasites counteradapt to overcome mechanisms of host resistance. B) As resistance spreads in a population, parasite prevalence will typically decrease (due to lack of suitable hosts), thus reducing the strength of parasite-mediated selection. If resistance carries a fitness cost, it should be selected against in this new environment, and susceptible host types will once again become common. C) If resistance is lost (either by mutation or phenotypic change) at a constant rate, the continual regeneration of susceptible host types in an otherwise resistant population can maintain a parasite population in an environment dominated by resistant host types.

The second way resistance can be lost from a host population is due to selection against costly host resistance (whereby resistant hosts tend to produce fewer offspring than susceptible hosts; [17]). In this case, high parasite densities in the environment can select for resistance initially, but as the number of parasites decreases due to the spread of resistant hosts, selection should act to reduce resistance in the host population, once again giving the parasites a population of susceptible hosts to infect [18]. This could eventually lead to an increase in parasite density (and disease prevalence) and therefore selection once again for host resistance. Such dynamic changes between host resistance and parasite infectivity are an example of an “ecological feedback,” in which the local ecology (in this case local parasite density) changes the strength of parasite-mediated selection on host populations, and thus the evolutionary response of host populations (in this case, selecting either for or against costly resistance). These dynamics are in contrast to the evolutionary cycling of host and parasite genotypes as a result of negative frequency-dependent selection, whereby parasites adapt to overcome resistance of common host types [3], as described in the previous paragraph.

The loss of resistance as a result of relaxed parasite-mediated selection assumes that resistance carries a significant fitness cost for hosts [17]. Costs associated with resistance have been uncovered across diverse host–parasite systems, most often by comparing the growth rate and/or fecundity of resistant and susceptible individuals in a parasite-free environment [19]. However, the magnitude and shape of these costs has proven hard to measure due in part to the context-dependent nature of host fitness [20]. In the case of bacterial resistance against phages, for example, costs associated with resistance via receptor modification have been well documented across systems and used to help explain microbial diversity [13]. However, costs associated with resistance will depend both on the mechanism of resistance and the environment in which the host exists; resistance of the plant pathogen *Pseudomonas syringae* to phage was found to decrease growth of the bacterium on its host plant but was found not to impact growth rate in vitro [20]. As such, predicting how rapidly host resistance will be lost due to selection requires information both about the strength of parasite-mediated selection in the local environment and the magnitude of fitness costs associated with resistance in that same environment. Evidence for loss of costly resistance exists across systems but so too does evidence for the maintenance of resistance in the absence of parasite-mediated selection [21], suggesting that this mechanism might not be generalizable across systems in describing the coexistence of hosts and parasites.

A third and more recently described mechanism by which resistance can be lost from a host population is due to host reversion to susceptibility [12,22]. In contrast to counteradaptation by parasites or selection against less fit resistant types, this explanation relies on the loss of immunity by either back mutation or phenotypic change in previously resistant host lineages. The key idea here is that parasites select for host resistance, as above, but that as host resistance spreads and even fixes in a host population, the parasite population can be maintained due to a small but substantial subpopulation of susceptible hosts that is continually spun off from the resistant host population (an idea put forward in part by Delbruck for bacteria–phage coexistence over 70 years ago; [23]). This newly susceptible host type will either be selected for (if resistance is costly and parasite-mediated selection is relatively weak; Fig 2A), it will “drift” (if selection is neutral), or it will be selected against (if parasite-mediated selection is strong; Fig 2B). In many ways, this idea mirrors that of waning immunity, and similar models have been proposed to describe how waning immunity after vaccination may hinder the eradication of disease from a host population [24].

**Fig 2:**
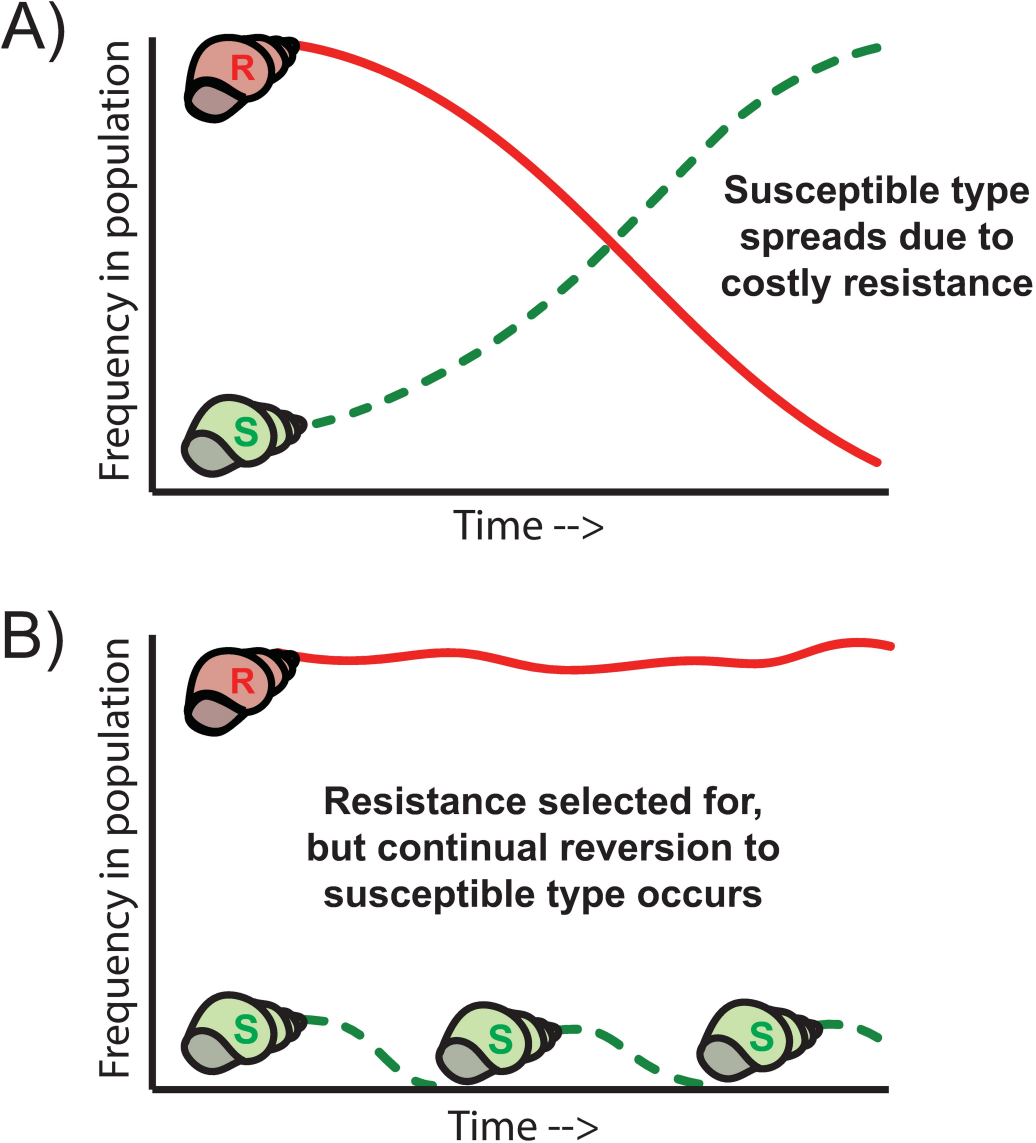
Examples of two possible evolutionary fates of a reversion mutant (a previously resistant genotype that has lost immunity) that arises within an otherwise resistant population. A) If parasite-mediated selection is relaxed and resistance is associated with significant fitness costs, the susceptible mutant should increase in frequency over time. B) If parasite-mediated selection is strong, susceptible mutants should be continually selected against and resistance maintained. However, if the loss of resistance occurs regularly and at a high enough rate, the population of susceptible types that are continually reintroduced could maintain an active parasite population, thus maintaining parasite-mediated selection over time even after resistance is “fixed” in the population.

Two pieces of recent evidence from bacteria–phage systems support the idea that loss of resistance via reversion to susceptibility is both possible and a likely explanation for bacteria–phage coexistence [12,22]. First, a study focused on CRISPR-Cas bacterial immunity (in which CRISPR sequences acquired within the bacterial genome as a result of previous unsuccessful phage infections confer a specific immune memory that protects the cell against future infections) suggested that loss of resistance could be a more likely mechanism to explain bacteria–phage coexistence than selection against costly resistance [12]. Experimental coevolution of *Streptococcus thermophilus* and its lytic phage 2972 was found to result in stable coexistence in a subset of experimental lines despite the seeming fixation of resistance (i.e., the acquisition and persistence of particular spacers over time). Results of theoretical exploration using both analytical and numerical analyses suggest that, unlike incorporating autoimmunity as a consequence of resistance, loss of CRISPR-Cas immunity is a more likely explanation for these results, as here the maintenance of phage populations was found to occur under the most realistic parameter space. This is in line with previous results from a related bacterium, *S*. *epidermidis*, which was found to lose phenotypic functionality of CRISPR-Cas immunity at a rate of 10^−4^–10^−3^ inactivation/loss events per individual per generation [25].

This result was recently extended to phenotypic and/or genetic transition from resistance to susceptibility in experimental populations of *Escherichia coli* coevolving with a virulent mutant of the model phage Lambda (λ^VIR^) [22]. In this case, a mass action model based on bacteria–phage dynamics in liquid culture was used to identify the conditions under which transition from resistance to susceptibility can stably maintain a phage population, and an estimated rate of 10^−5^ reversions per cell per hour or greater was suggested as the base line for this mechanism to work. Importantly, the model also indicated that loss of resistance due to fitness costs was unlikely to maintain bacteria and phage coexistence in this system, as the fitness costs associated with resistance that would be necessary for maintaining stable phage populations would be very high (>20%). The model was experimentally validated by coculturing a population of resistant hosts (i.e., those initiated from one of 12 independent resistant mutants) and phage and observing that, in eight out of 12 cultures, phages were maintained despite their inability to infect the resistant type. This variability among lines likely reflects variation in resistance mechanisms that evolved and suggests that resistance-to-susceptible transitions will occur at different frequencies depending on the resistance mechanism in the population. To further test whether the theoretical threshold reversion rate was attained in this system, a genetically marked temperate phage Lambda (λ^KAN^) was used to indicate the frequency of susceptible bacterial cells in the population. Here, susceptible cells that were infected by the phage and converted to lysogens (in which the phage integrates into the bacterial genome) were resistant to the antibiotic kanamycin and therefore could be counted separately from resistant cells, a very clever experimental trick that was critical to testing the central hypothesis. Indeed, the estimated rate of lysogen production (a surrogate for reversion to susceptible type) was found to be higher than that predicted to be required by the model for the majority of mutants tested, and the rate of lysogen production was predictive of phage persistence in coculture for a given mutant. This result in itself was surprising, given the remarkably high rate of reversion theoretically required to maintain the phage population. Together, these results strongly support the hypothesis that bacteria–phage coexistence can be explained in part by the spontaneous loss of resistance in a resistant bacterial population.

One important aspect of this newly described loss of bacterial resistance is that it helps explain how phage-mediated selection might be maintained over time. In contrast to more simple models in which ecological feedbacks would lead to selection against costly resistance once the parasite pressure dissipated, this model predicts that the continual regeneration of susceptible hosts maintains the parasite population and allows constant selection for resistant hosts. However, the generality of this model to other parasite (or even phage) systems will likely depend on both the frequency of transitions from resistance to susceptibility and how many secondary infections are produced from a single successful infection (a parameter known as R0 in disease ecology). For example, if the parasite has a low transmission rate, such that either few propagules are produced within each infected host or the likelihood of propagules reaching another individual is low, then the likelihood of transmitting to a rare susceptible host (i.e., one that has had a reversion mutation and thus lost resistance or else is a progeny of this host) would be low, and the parasite population would likely not persist under this model. Furthermore, the rate of reversion from resistance to susceptible will almost certainly depend upon the mechanism of resistance that evolves (as suggested across the 12 bacterial lines used in [22]) and therefore could be variable within a single host–parasite system as well as among systems. Thus, moving forward, there is a clear need to determine both how common reversion to susceptibility is within/across systems and how important this mechanism is in maintaining parasite reproduction in predominantly resistant host populations. This could include, for example, addressing the importance of other demographic, evolutionary, and ecological variables such as population size, spatial structure and refuges, host migration rate, and/or parasite diversity.

## Abbreviations

CRISPR: clustered regularly interspaced short palindromic repeats
RM: restriction modification
*RPP13*; λ^KAN^: temperate phage Lambda
λ^VIR^: model phage Lambda
